# Medical and Philosophical Intelligence

**Published:** 1812-02

**Authors:** 


### MEDICAL AND PHILOSOPHICAL INTELLIGENCE.

### ROYAL SOCIETY.

NOV. 28, and *Dec.* 5.—The conclusion of Mr. Brande's researches on the Blood read. The result of the author's experiments is, that very little iron exists in the blood; that the quantity is so small as to render it very improbable that the color of that fluid depends on it; and that its influence is much less than has been supposed.

*Dec.* 12. A paper by Mr. Home, on the Structure of the Ear of the Whale-bone Whale (*Balena mysticeta*) was read, in which is described the situation and dimension of the tympanum and adjoining parts.

*Dec.* 19. The first part of a paper by Dr. Herschell on the Comet was read. Dr. H. noticed something like a distinct luminous body about the centre of the nucleus, which changed its relative position, sometimes appearing nearer, at others further, from the side next the sun; and differing, under these circumstances, very much in brilliancy. From these facts he was led to infer, that the comet enveloped a real planetary body; and, after a series of observations, on the 16th of October, when the comet was 114 millions of miles from the earth, he ascertained that this body was 428 miles in diameter, and surrounded with a cometic atmosphere. For this purpose he viewed it with seven, ten, and twenty, feet telescopes, containing magnifiers of various powers, from 40 to 600 times.

---

A new comet has just been seen (beginning of December) in the constellation of Eridanus, by M. Pons, at Marseilles, on the 16th of November, but the thickness of the weather prevented its being visible at Paris until the 6th of December. It is not visible to the naked eye; but with very good glasses its nucleus, the light of which is vivid, is seen surrounded with a light cloud, but is without the slightest trace of a tail. On the evenings of the 22d and 23d of December it was seen from the observatory at Glasgow.

---

*Case of Abortion from diseased Structure of the Placenta.*—Mrs. E., a very delicate and hysterical woman, about twenty years of age, miscarried twice in one year, and shortly afterwards became pregnant again. By very rigid adherence to the proper plan of treatment, she passed over the third month, the period of her former abortions, and there was every reason to hope that she would go safely to her full time. Between the fifth and sixth month she ceased to feel the movements of the fœtus, excepting slightly, and at long intervals, and between the sixth and seventh month, she was seized one evening with severe uterine pains, and the appearance of a colored discharge; the pains continued all night, became more violent towards morning, when a fœtus, completely enveloped in its membranes, was expelled. On rending open the ovum, the fœtus appeared to have been dead a considerable time, and, on turning ing up the placenta, its whole structure was found to be thickly studded with tubercles, differing in size from a pea to a filbert. This diseased formation of the placenta must have impeded the passage of nourishment from the mother to the fœtus, and thus occasioned the death of the latter, and its consequent expulsion from the uterus.—*Lond. Med. Rev.*

---

Comparative Statement of the Population of England, Scotland, and Wales, in 1801 and 1811; now before the House of Commons.

| | POPULATION IN 1801. | | |
|---|---|---|---|
| | Males. | Females. | Total. |
| England - - - - - - | 3,987,935 | 4,343,499 | 8,331,434 |
| Wales - - - - - - - | 257,178 | 284,368 | 541,546 |
| Scotland - - - - - - | 734,581 | 864,487 | 1,599,038 |
| Army, Navy &c. - - | 470,598 | - - - - | 470,598 |
| Totals - - | 5,450,292 | 5,492,654 | 10,942,646 |
| | POPULATION IN 1811. | | |
| England - - - - - - | 4,555,257 | 4,944,143 | 9,499,400 |
| Wales - - - - - - - | 289,414 | 317,966 | 607,380 |
| Scotland - - - - - - | 825,377 | 979,487 | 1,804,864 |
| Army, Navy, &c. - - | 640,500 | - - - - | 640,500 |
| Totals - - | 6,310,548 | 6,241,596 | 12,552,144 |

| INCREASE. | |
|---|---|
| England - - - - | 1,167,966 |
| Wales - - - - - - | 85,834 |
| Scotland - - - - | 208,180 |
| Army, Navy, &c. - | 169,902 |
| Total - - | 1,811,880 |

A supply of wholesome provisions, is a principal means by which health is preserved or restored. The immense population of London requires a supply of food not only extensive but regular. With regard to the article of fish, the supply has been considered as defective; but this promises to be remedied by the establishment of an iron railway from the metropolis to the coast of Essex, by which fish will be brought at all times speedily, certainly, and at a smaller expence, to the markets. A prospectus of this project has been published lately, though we believe not generally known.

---

A regular silver vein has been found just on the Cornish side of the river Tamar. Although small quantities of this metal have frequently been got, in cross veins, in the mines of Cornwall, yet no regular silver lode has ever before been met with. This vein was found and traced from the surface, and is now regularly worked as a silver mine. The operations are still very recent; and it is only within a very short time that enough of the metal has been got to render it worthy of observation. This lode is in Killow, the Schiston rock of Cornwall, and runs nearly parallel to two copper lodes which are near it, the one on the north, the other on the south, side. At the surface the vein chiefly consists of the clayey matter, denominated *flookau*, which is mixed with the earthy black ore of silver; deeper, native silver, with red silver ore; and at the greatest depth, which is about twenty fathoms, the red ore is found more compact, along with vitrious silver ore. These lie chiefly on spathone iron ore, and are mixed with arsenical pyrites.

*On the relative State of the Barometer and Thermometer at the foot and on the summit of Skiddaw.*—The morning (Oct. 28th, 1811) was cloudy and likely for showers, but, this being the last day that I could conveniently stop in Keswick, I resolved to attempt the ascent of Skiddaw. In the room where I breakfasted, the thermometer stood at 58°; another in the open air out of doors stood at 55°. The barom. at Keswick stood at 29·088 inches. At Mr. Calvert's the barom. was at 29·1 inches, and the thermom. at 55°, the same as at Keswick. These observations were made upon the terrace before Mr. Calvert's door. At the top of Skiddaw the barom. stood at 26·2 inches, and the thermom. at 40°. We had three thermometers, all of which were as near 40° as could be perceived. On our return, on Jenkin Hill, the barom. stood at 26·9 inches, and the thermom. at 44° 5. At a spring nearly a hundred yards below Jenkin Hill the barom. stood at 27·2, and the thermom. in the air, on the ground, and in the water, viz. the spring, as follows: in the air 43° 5, on the ground 42°, and in the water 41°; all the thermometers agreed here as well as upon the top of Skiddaw. On the opposite side of the hill to the spring just mentioned, at another spring, the barom. stood at 27·2, and the thermom. in the water at 43°, in the air at 42°, on the ground the same as in the air. At another place, the name of which I have forgotten, where the barom. stood at 27·8 inches, the thermom. was at 45°. At the spring above the High, the barom. stood at 27·89, and the thermom. in the air at 46·25, in the water 44°, and on the ground 44°; but the distance above 44° was so small as not easily to be expressed. At a spring near Long Scale Gate the barom. stood at 28·22; the thermom. in the air at 47° 5, in the water 48°, on the ground 48°. At a spring on the opposite side of the hill to Long Scale Gate, the barom. stood at 28·3; and the thermom. in the air, in the water, and on the ground, stood at 48°. When we got back to Mr. Calvert's, the barom. and thermom. stood as in the morning, viz. 29·1 inches the barom. and the thermom. at 55°. On examining Mr. Calvert's barom. in a back room where there had been no fire, or any thing to influence the alteration of the height of the mercury, save the weight of the atmosphere, the barom. had rather risen than otherwise. The height of Skiddaw from Keswick is 930 yards; Jenkin Hill 695; the spring before mentioned, where the barom. stood at 27·2, and the thermom. in the air at 43°, on the ground 42°, in the water 41°, 600 yards; and the top of Laterigg 296 yards. We left Mr. Calvert's about ten o'clock in the forenoon, and returned about half-past four in the afternoon. There was not much rain till after three o'clock. The wind was exceedingly strong upon the top of Skiddaw, and the sensation the cold produced, produced, equalled, if not exceeded, any I ever experienced. The party consisted of Mr. Calvert, Mr. Otley, and myself; and, though the thermom. was not below 40°, all of us felt extreme cold sensations. There was no rain during the time we were upon Skiddaw. According to Donald, Skiddaw is 958 yards above the level of Bassenthwaite Lake. Keswick is considerably higher than this lake; hence it is probable that both calculations were accurately made. Paris Mountain in Wales is 3720 feet high, and the mercury varied from the bottom to the top of this hill 3·75. By comparing these experiments together, we shall find, that for every inch that the mercury sinks in the barometer tube, there will be nearly 1000 feet of elevation. It will fall a little short of 1000 feet for an inch of mercurial depression. We find that there was no sensible variation in the temperature, from the time we left, to the time we returned, to Mr. Calvert's. We likewise find that the thermom. was 15° lower upon the top of Skiddaw, than at Mr. Calvert's. If we divide the number of yards which the hill is high, by the number of degrees which the thermom. sinks, we shall have the number of yards for each degree of the thermometrical depression, viz, 930 ÷ (the height of Skiddaw) 15 = 62, so that there will be 62 yards of elevation for every degree that the thermom. sinks. When the mercury in the barom. falls an inch by ascending an hill, we may calculate nearly 1000 feet; and when the thermom. sinks a degree, we may reckon 186 feet of elevation.

---

*Case of Vaccine, and of Chicken Pox, occurring together in the same Patient.*—Mrs. Morley, of Gardner's-lane, applied at the Western Dispensary to have her child *vaccinated*, which was done on the 2d of December. Nothing material occurred until the 9th, when Mr. Furnival, the apothecary, was requested late in the evening to see the child, which he understood was dying. He found it much convulsed. On the following morning an eruption appeared, which bore much the resemblance of the small-pox. The validity of this supposition was supported by the disease having existed in the next room for a fortnight, without the parent's knowledge. On the 14th the vaccine disease was very distinct, although surrounded by many of the pustules in a state of maturity; they were, however, very small. Prior to the appearance of the eruption, the after-mentioned patient (Mrs. Winsall's child) was vaccinated from the arm; and, on the 16th, Henry Kite was inoculated from some of the eruptive pustules in the foot. At this time they all appeared to have desquamated, and only the arm where the vaccine virus was inserted shewed the remains of the disease; which was rather singular, as one of the scabs, the usual remnant of the disease, was elevated in a conical form to the height of nearly a quarter of an inch, which symptom now gradually disappears, but the child is indisposed with a complaint in the eyes, slight fever, and general debility. Dr. Blegborough and Dr. Fothergill, who saw the child at different periods of the eruption, regarded it as the chicken-pock.

Mrs. Winsall, of Charles-street, had her infant vaccinated from the above patient on the 9th of December, in the morning. The disease appeared to have taken on the 13th, and on the 18th clearly evinced the vaccine virus to have taken its proper effect; since which time the child has not been seen.

On the 16th of December, Henry Kite, of New Peter-street, was inoculated from one of the pustules on the foot of Mrs. Morley's child, being the only one that then appeared to have any fluid in them. On the 21st there was hardly any indication of the success of the attempt, but on the 23d a pustule with the vaccine character, having a small degree of inflammation around it, was evident. On the 26th the pustule contained a small quantity of fluid, and the inflammation was increased. On the 28th the symptoms were not so high as before, and the disease appeared on the wane. The child did not evince any illness, nor has any eruption appeared in other parts of the body. He has since been inoculated with variolous matter, and the disease occurred in the usual course, in a mild but unequivocal form.

---

We are requested to mention that Dr. Irvine, whose book upon the Diseases of Sicily we reviewed in our last number, has fallen a sacrifice to his professional exertions. He died at Malta, of a fever caught during his attendance upon some French prisoners; and has left a widow and five young children, without any support. Dr. Irvine had proposed to write an account of Sicily; and a sketch of this work, found after his death, is now preparing to be published for the benefit of his family.—Messrs. Thomas Coutts and Co. Strand, and Mr. Mawman the publisher, No. 39, Ludgate-street, have kindly consented to receive subscriptions for the publication, which, independently of its claims upon the benevolence of the public, is, we understand, a work of considerable interest and merit.

---

Dr. Cheyne, late of Leith, and now of Dublin, has in the press, and nearly ready for publication, an extensive work on Apoplexy, with plates illustrative of that disease.

---

Dr. Reid will commence his next course of Lectures on the Theory and Practice of Medicine, on Monday the 10th of February, at nine o'clock in the morning, at his house, Grenville-street, Brunswick-square; where the course will be continued at the same hour, on Mondays, Wednesdays, and Fridays, until its conclusion.

---

Dr. Hooper and Dr. Ager will recommence their Lectures and Examinations on the Theory and Practice of Physic, Chemistry, and the Materia Medica, at the Theatre, 26, Cork-street, Burlington-gardens, on Monday, February 3d, 1812, at eight o'clock in the morning. Three courses are given yearly, beginning in February, June, and October, each on the first Monday of the month.

---

Mr. Moor, Surgeon-Dentist to her Royal Highness the Dutchess of York, will commence a course of Lectures on the Structure and

Diseases of the Teeth, on the 6th of February, in which will be explained the complete practice of the dentist.

---

Mr. Singer and Mr. Lydiatt are delivering their course of Lectures in the following order:

Saturday, Feb. 1st.—Mr. Lydiatt. Mechanic Arts. Lecture 9th. —Nature and properties of mercury; its application to the arts, &c.

Tuesday, Feb. 4th.—Mr. Singer. Natural Philosophy. Lecture 10th.—Pneumatics. Condensation of air. Phenomena resulting from this process. Effect of condensed air on sound, and on combustible bodies.

Saturday, Feb. 8th.—Mr. Lydiatt. Mechanic Arts. Lecture 10th.—Explanation of the properties and combinations of tin, lead, and bismuth.

Tuesday, Feb. 11th.—Mr. Singer. Natural Philosophy. Lecture 11th.—Phenomena of the atmosphere. Cause of wind. Nature of the rainbow, haloes, and various optical illusions.

Saturday, Feb. 15th.—Mr. Lydiatt. Mechanic Arts. Lecture 11th.—Mechanical construction of locks. Principles on which their security depends.

Tuesday, Feb. 18th.—Mr. Singer. Natural Philosophy. Lecture 12th.—Phenomena of dew, rain, hail, and snow. Origin of springs and rivers. Prognostication of changes in the weather. Effect of the atmosphere on the constitution and manners. Summary, and conclusion of the course.

Saturday, Feb. 22d.—Mr. Lydiatt. Mechanic Arts. Lecture 12th.—Continuation of the principles of lock-work. Explanation of several ingenious contrivances in this branch of manufacture. Conclusion of the course.

